# Pressure-sensitive liquid phase epitaxy of highly-doped n-type SiGe crystals for thermoelectric applications

**DOI:** 10.1038/s41598-019-39786-y

**Published:** 2019-03-13

**Authors:** Hung-Wei Li, Chih-Wei Chang

**Affiliations:** 1 Center for Condensed Matter Sciences, National Taiwan University, Taipei, 10617 Taiwan; 2 Center of Atomic Initiative for New Materials (AI-MAT), National Taiwan University, Taipei, 10617 Taiwan

## Abstract

Based on recent works, the most desirable high-temperature thermoelectric material would be highly-doped Si_1−x_Ge_x_ crystals or films with sufficiently high Ge concentrations so that simultaneous enhancing the power factor and wave-engineering of phonons could be possible on the ballistic thermal conductor. However, available thin film deposition methods such as metal organic chemical vapor deposition, electron-beam evaporation, or sputtering are unable to produce highly-doped SiGe single crystals or thick films of high quality. To fabricate the desired material, we here employ liquid phase epitaxy to make highly-doped (up to 2% GaP doping) SiGe crystals with minimized concentration variations on Si (111) and (100) substrates. We find that growing Si_1−x_Ge_x_ (x = 0.05~0.25) crystals from Ga solvents at relatively high vacuum pressure (0.1 torr) displays significant deviations from previous calculated phase diagram. Moreover, doping GaP into SiGe is found to affect the solubility of the system but not the resulting Ge concentration. We thus plot a new pressure-dependent phase diagram. We further demonstrate that the new pressure-induced liquid phase epitaxy technique can yield Si_1−x_Ge_x_ crystals of much higher Ge concentrations (x > 0.8) than those grown by the conventional method.

## Introduction

Recent experimental discoveries of room temperature ballistic thermal conduction have opened possibilities for engineering wave properties of phonons^1,2^. Particularly, the ballistic thermal conduction is found to persist over 8 μm at 300 K in homogeneously-alloyed SiGe nanowires^1^. The unprecedented microscale ballistic thermal transport would reduce many technical barriers for unraveling many interesting phenomena. The phonons carrying out heat conduction in SiGe are estimated to occupy only 0.04% of the excited phonon spectrum, and most other short-wavelength phonons are localized due to alloy scatterings^1^. Hence, although the thermal conductivity of the SiGe nanowires is low (<10 W/m-K), the phonon mean free path is much longer than that of Si nanowires.

On the other hand, SiGe are known to be thermoelectric materials with superior figure of merit (*ZT* = *σS*^2^*T*/*κ*, here *σ* is electrical conductivity, *S* is themopower, *κ* is thermal conductivity, and *T* is temperature) at high temperatures (*ZT*~1 at *T* = 1200 K). The *κ* of Si_1−x_Ge_x_ is known to drop very quickly as x increases and the *ZT* is predicted to reach maximum at x = 0.5^3,4^. The microscale ballistic thermal conduction, combined with the thermoelectric properties, would make SiGe useful for realizing next-generation thermoelectric devices inspired by phononic crystals^5^.

Because the dominant low-frequency phonons carrying out the heat conduction are estimated to have wavelengths longer than 10 nm, which is much shorter than the diameters (~200 nm) of SiGe nanowires, the observed ballistic thermal conduction is suggested to be a bulk property of SiGe^1^. Indeed, even though each material has their own *ZT* characteristics at different temperatures and different synthetic strategies have been applied to enhance *ZT*^6,7^, so far SiGe is a unique material demonstrated to exhibit ballistic thermal conduction for a wide range of alloy concentration. As mentioned above, strong length dependent thermal conductivities have been found in Si_0.9_Ge_0.1_ and Si_0.4_Ge_0.6_ nanowires^1^. Thickness dependent thermal conductivity has also been found in Si_0.9_Ge_0.1_~Si_0.7_Ge_0.3_ superlattice films^8,9^. Rowe *et. al*. discovered that when the N-type, heavily-doped (~10^20^/cm^3^) Si_0.63_Ge_0.26_ grain size is less than 5μm, 28% reduction of thermal conductivity is observed^10^, indicating ballistic thermal conduction remains robust even when electron-phonon interactions could enhance scatterings of low-frequency phonons^11^. Furthermore, calculations based on density functional theory suggests that ballistic thermal conduction should be longer than 1 µm^12^. Frequency dependent time-domain thermal reflectance measurements on bulk Si_0.4_Ge_0.6_ also suggest pronounced contributions from ballistic phonons^13^. These property indicates that large area applications based on the novel ballistic thermal conduction could be realized in SiGe crystals or thick films of microscale thickness. Yet, to experimentally investigate their ballistic thermal conduction, SiGe crystals or films of micrometer thick will be needed. To further realize their thermoelectric applications, highly-doped SiGe films are also required. Unfortunately, commercially available SiGe wafers usually contain Ge concentration less than 10% and cannot meet the unusual doping requirement. In addition, many available thin film techniques, such as metal organic chemical vapor deposition, electron-beam evaporation, or sputtering often yield polycrystalline films. Besides, many post-doping processes are unable to produce highly-doped SiGe films required for optimizing the thermoelectric performance.

Enhancing thermoelectric performance of SiGe by GaP doping was found to give 20~30% improvement of *ZT* compared to standard P-doping. Vining and Fleurial concluded that the enhancement can be attributed to an increase of carrier concentrations from 2.1 × 10^20^ cm^−3^ (0.4% P doped) to 3.3×10^20^ cm^−3^ (0.4~2% GaP doped) that leads to an improved power factor^14^. Grain size effects, which were considered to reduce *κ*, were suggested to be minimal^14^. Yet, recent works employing 2.5% GaP doping on SiGe nanoparticles of 13 nm diameters was found to reach *ZT* = 0.95 at 1200 K and a ten-fold reduction of *κ* was observed^15^. Although the subtleties of increased carrier concentrations and reduced thermal conductivity have not been fully understood, these works indicate the possibilities for further engineering *σ* and *κ* in SiGe.

To exploit the ballistic thermal conduction properties in GaP-doped SiGe, high quality crystals will be needed. However, the quaternary compound cannot be easily made by the common thin-film deposition methods. Liquid phase epitaxy (LPE) technique is ideally suited to make the desired highly-doped, single-crystalline SiGe films and crystals. In recent years, LPE has been investigated for applications from solar cell, thermoelectric devices, and to customized epitaxial substrate structures. In particular, the low capital equipment and operating costs; the absence of toxic precursors or byproducts; and producing Si layers with detect densities an order of magnitude lower than that of the Si substrates are the major advantages. Recently, LPE is found to be especially suited for conducting epitaxial lateral overgrowth on patterned or masked substrates for novel device structures^16,17^.

SiGe crystals or films grown by LPE methods were reported starting in late 1980 s^17–28^. Various solvents including, In^18^, Bi^20^, AuBi^29^, Sn^24^, and Ga^24^ have been used. Good agreements were found between the calculated phase diagram and the ternary systems using Bi as solvents^20^. However, comparing to the LPE used for making undoped or normally doped SiGe films, literatures on highly-doped SiGe films are scarce. Borshchevsky and Fleurial mentioned unsuccessful attempts to grow SiGe out of Ga-P melts^24^. They attributed it to complex interactions in the quaternary system. Helped by further adding In into the Ga solvent, they reported successful doping GaP into Si_0.8_Ge_0.2_ crystals by LPE and found that the Ga additions can significantly enhance the P solubility and carrier concentration. In addition, the crystals grown by LPE have lower resistivity than those made by hot-pressed methods. In light of the recent interests in exploring ballistic thermal conduction and its potential applications in thermoelectrics, we find that many of the previous LPE growth conditions and material characterizations of GaP-doped SiGe cystals or films should be clarified. In this paper, we particularly investigate effects that were overlooked by previous works.

**Figure 1:**
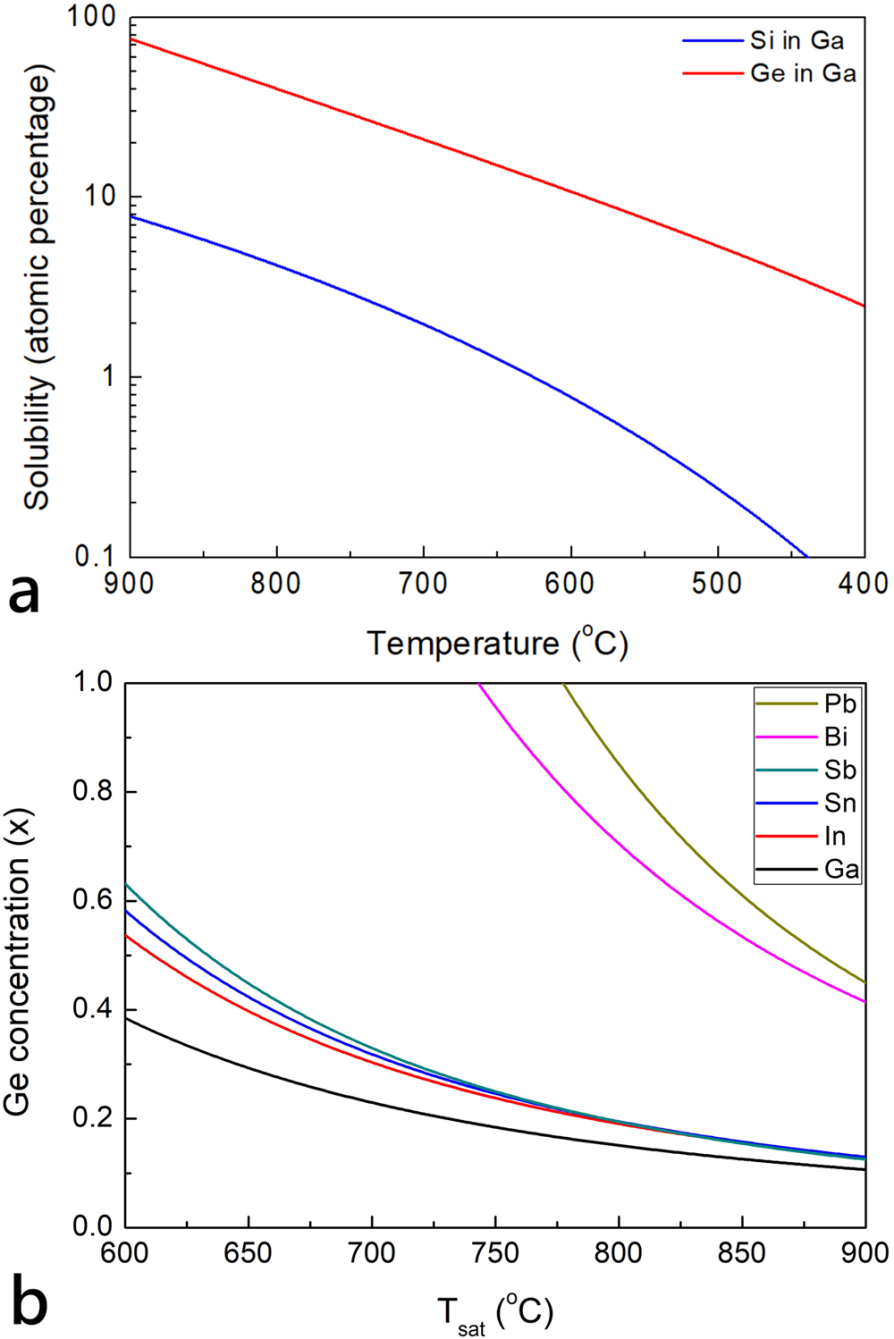
(**a**) Solubility vs. temperature for Si in Ga (blue curve) and Ge in Ga (red curve). (**b**) Calculated Ge concentration vs. T_sat_ for different solvents using Eq. (1) with *x*_*liquid*_ = 0.1 and the parameters listed in ref.^31^.

The binary solid solution of Si and Ge in various solvents are shown in Fig. 1(a). We see that the solubility of Ge the metal solvents are much more than that of Si. The ternary phase diagram of SiGe in various solvents is obtained from thermodynamic equilibrium model of Malmejac *et al*.^30^, following Thurmond and Kowalchick’s work on binary Si-metal or Ge-metal systems^31^. From the model the Ge concentration (x) in the solid phase of Si_1−x_Ge_x_ can be expressed as a function of the mole fraction in the liquid phase (*x*_*liquid*_) at saturation temperature (T_sat_):

1 $${\rm{x}}\approx \exp \{\frac{1}{R{T}_{sat}}[{\rm{\Delta }}{H}_{F,Ge}(1-\frac{{T}_{sat}}{{T}_{F,Ge}})+{\lambda }_{Ge,solvent}]\}{x}_{liquid}$$

Here *ΔH*_*F,Ge*_ = 8830 cal/mole is the latent heat of Ge for fusion, *T*_*F,Ge*_ = 1210 K is melting point of Ge, and *λ*_*Ge,solvent*_ = −150 is the interaction parameter in different solvents^20,31^. As shown in Fig. 1(b), for a fixed T_sat_, Bi or Pb favors Ge-rich samples, whereas In or Ga leads to Si-rich samples. Because our goal is to make highly-doped SiGe crystals, we will start from growing undoped SiGe using Ga solvent and then further employing similar conditions for GaP doping.

**Figure 2:**
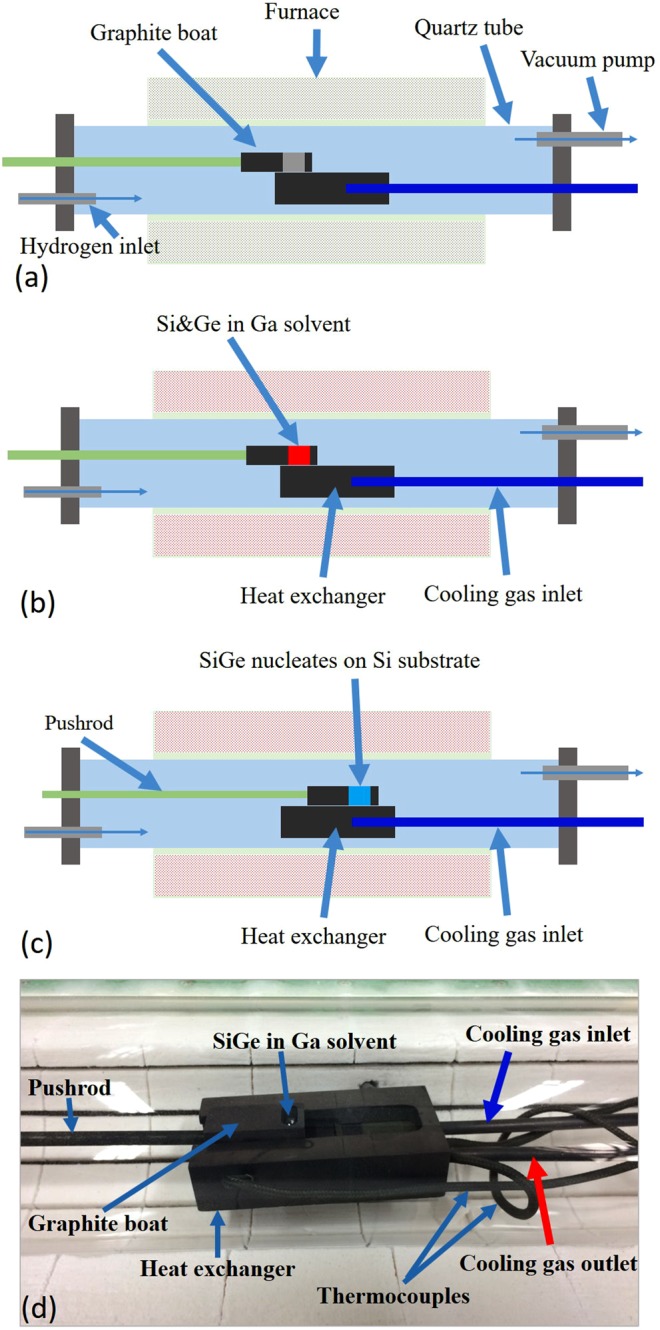
Schematics of LPE procedure (**a**) A quartz tube was evacuated and then continuously purged with H_2_ before raising the temperature of the furnace. (**b**) After reaching the highest set temperature (T_high_ = T_sat_ + ΔT), the temperature was held for more than 2.5 h to homogenize the solution. We then turned on the cooling gas so the heat exchanger and the Si substrate were cooled down to T_sat_. (**c**) Using a pushrod to slide the boat and nucleation of the seeds started. The growth time varied from 1.5 to 18 hours. Finally, the growth was terminated by removing the solution away from the substrate (not shown). (**d**) A photo of the LPE assembly inside a furnace.

A tipping boat method was usually employed in earlier works. But, as will be shown later, we found that there were always large amounts of residues sticking to the substrate and could not be easily removed. On the other hand, the sliding boat method, which involved graphite crucible designed for efficient heat exchange at the substrate and *in-situ* temperature monitoring, offered better controllable growth conditions. As shown in Fig. 2(a,b), here a home-made heat exchanger was used for cooling the substrate via pressurized gas with regulated flow rates. The temperature of the substrate was simultaneously monitored by three thermocouples located at different positions during the entire process. We had also varied the cooling rates from −1 °C/min to 0 °C/min during the growth. Unlike the tipping boat method, only a thin layer of residues was found in the sliding boat method and could be peeled off from the substrate after cooling it to room temperature. The grown crystals/films were characterized by energy dispersive X-ray spectroscopy (EDS), electron probe microanalysis (EPMA) equipped with wavelength dispersive spectroscopy (WDS), and x-ray electron spectroscopy (XPS).

## Results and Discussion

We firstly employ EDS to investigate concentration profiles along the thickness of SiGe crystals/films grown by different LPE methods and cooling rates. For crystals grown on Si (111) surface using the tipping boat method, a thick layer of Ge precipitates is found, whose morphology looks very similar to previous reports and could be mistakenly regarded as SiGe films in early works. Apparently, the unwanted Ge layer comes from residues that stick to the substrate mentioned above that cause the Ge precipitation at low temperature after the furnace is cooled. Cross-sectional EDS and mapping indeed displays that the Ge concentration varies abruptly along the thickness. Because the tipping boat method lacks the desired controllability during the growth, we have instead employed sliding boat method later.

**Figure 3:**
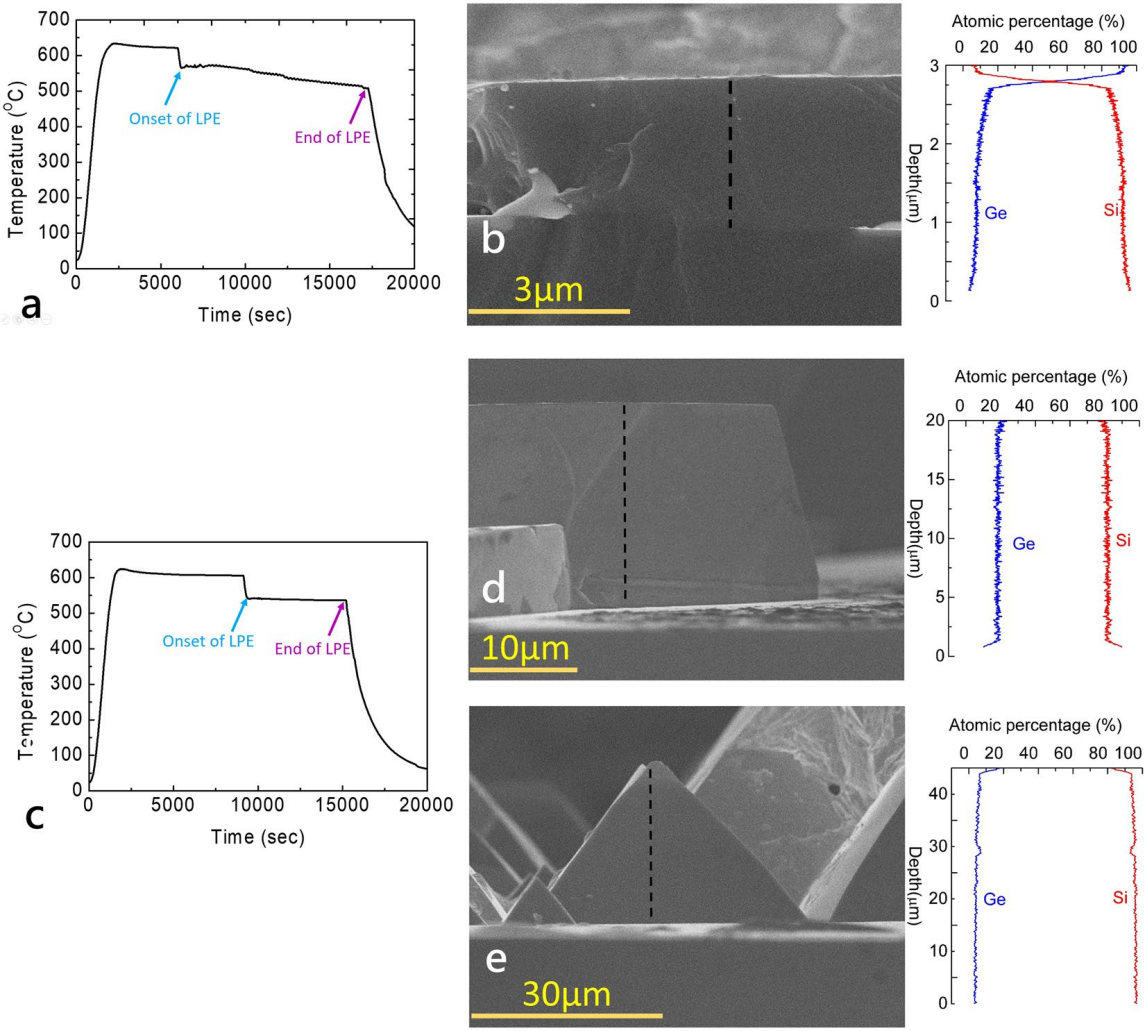
Temperature of a Si substrate vs. time during the LPE process. Here a cooling rate = −0.32 °C/min is employed. (**b**) A cross sectional SEM image of the grown SiGe crystal and the corresponding concentration profile determined by EDS. (**c**) Temperature of a Si substrate vs. time during the LPE process. Here a cooling rate = 0 °C/min is employed. (**d**,**e**) Cross sectional SEM images and the corresponding concentration profiles of the SiGe crystals grown on (**d**) Si (111) and (**e**) Si (100) surfaces, respectively.

Presumably to compensate the material loss in the solvent, a cooling rate with a gradually declined temperature during the growth was often employed by many previous sliding boat methods. Using a cooling rate = −0.32 °C/min shown in Fig. 3(a), the cross-sectional EDS shown in Fig. 3(b) displays the concentration profile of a representative grown crystal. We see that the Si concentration gradually decreases with thickness, whereas the Ge concentration steadily increases from 2% at the bottom to 15%, and then abruptly increases to 90% at the top of the crystal. Even though the top layer of Ge can be polished away, we find that there are always gradients of Si and Ge concentrations varying along the thickness of crystals whenever a non-zero cooling rate is employed. Because the Ge loss in the solvent is negligible in our experiment, we believe that the concentration gradient is due to the non-constant cooling rate that induces the increasing Ge concentration with increasing crystal thickness.

To minimize the concentration gradient, we have instead applied a fixed cooling rate = 0 °C/min during the growth. As shown in Fig. 3(c), here both the temperature of the furnace and the flow rate of the cooling gas are regulated to keep T_sat_ constant. Figure 3(d) displays that the grown Si_1−x_Ge_x_ crystal on a Si (111) substrate exhibit a much reduced concentration variation (Δx = ±0.007) even when the crystals are more than 20 μm thick. Further EPMA-WDS mapping on another crystal has confirmed the result (see supplementary information).

We have also applied identical growth conditions on Si (100) surfaces. Here the grown SiGe crystals are found to be of pyramidal shapes, as shown in Fig. 3(e). According to Sembian *et al*.^25^, the pyramidal growth is the result when the Ge concentration increases beyond 13%. Three-dimensional nucleation and growth have been suggested to be associated with the lattice mismatch between the grown layer and the substrate (lattice mismatch for Si/Ge = 4.2%) as well^18^. The pyramidal crystals grown on Si (100) sometimes give inhomogeneous Ge concentration distribution within a crystal. The inhomogeneity could originate from the uneven mixtures of Si/Ge in the solvent. Elongating the holding time at T_high_ from 2 to 8 hours indeed minimizes the problem. To reduce the concentration variation along the thickness, we again employ a constant cooling rate during the growth. The concentration variation is minimized, as shown in Fig. 3(e).

**Figure 4:**
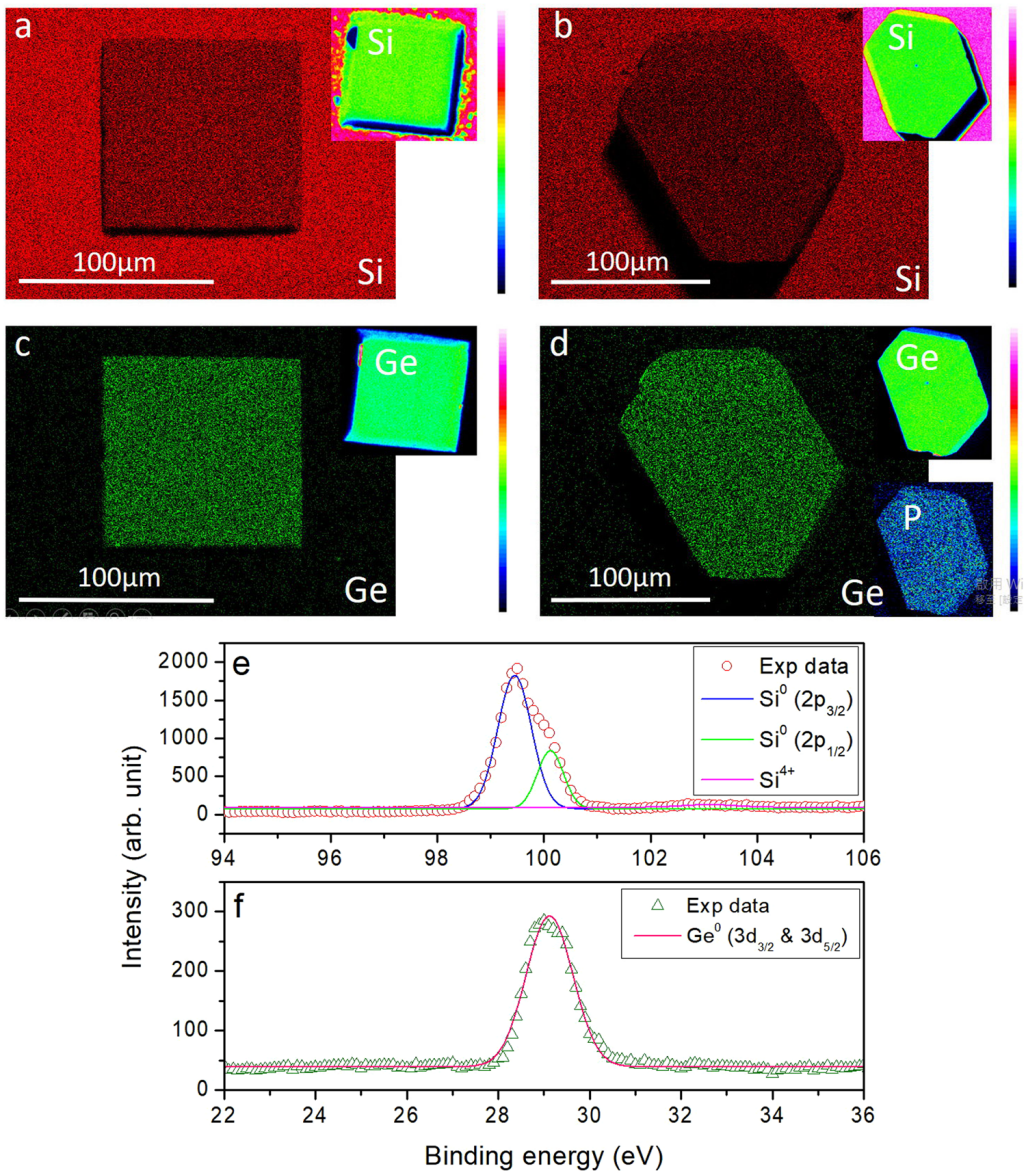
Representative EDS mapping of Si (**a,b**) and Ge (**c,d**) distributions of GaP-doped Si_0.84_Ge_0.16_ crystals grown on a Si (100) substrate (left images) and a Si (111) substrate (right images). The corresponding elemental mappings of EPMA-WDS are shown in the insets, further confirming the Si and the Ge are homogeneously distributed. Here the pyramidal crystal grown on Si (100) has been polished. The P concentration (0.5%) determined by EPMA-WDS shown in the inset of (**d**) also displays homogeneous distribution. (**e,f**) Representative XPS spectra of (**e**) Si 2*p* region and (**f**) Ge 3*d* region of the grown samples.

After the successful growth of SiGe crystals, we then proceed to do GaP doping. Interestingly, introducing 0.06~0.4% molar fraction (with respect to that of Ga) of P powder is found to significantly perturb the solubility of Si and Ge in Ga, and tiny SiGe crystals are found even when identical growth conditions mentioned above are applied. To solve the problem, we have followed Borshchevsky and Fleurial’s method and added a small amount (0.18~1.2% molar fraction respect to that of Ga) of In to the system^24^. However, differing from Borshchevsky and Fleurial’s work, we find that higher T_high_ is needed to dissolve the Si and Ge during the growth. The P concentration, as determined by EMPA-WDS, is found to reach more than 0.5%. The Si_0.84_Ge_0.16_ crystals grown on Si (100) and (111) substrates are also homogeneously alloyed, as shown in Fig. 4(a–d) by EDS and EMPA-WDS mappings.

The samples are further characterized by XPS of core level electrons in Si and Ge. As shown in Fig. 4(e,f), here the XPS spectra are deconvoluted to the doublet of Si 2*p*_3/2_ and 2*p*_1/2_ as well as Ge 3*d*_3/2_ and 3*d*_5/2_. Except that there is a small amount of detectable peak at 102.6 eV indicating the presence of SiO_x_ on the surface, no other bindings are found in the grown samples.

**Figure 5:**
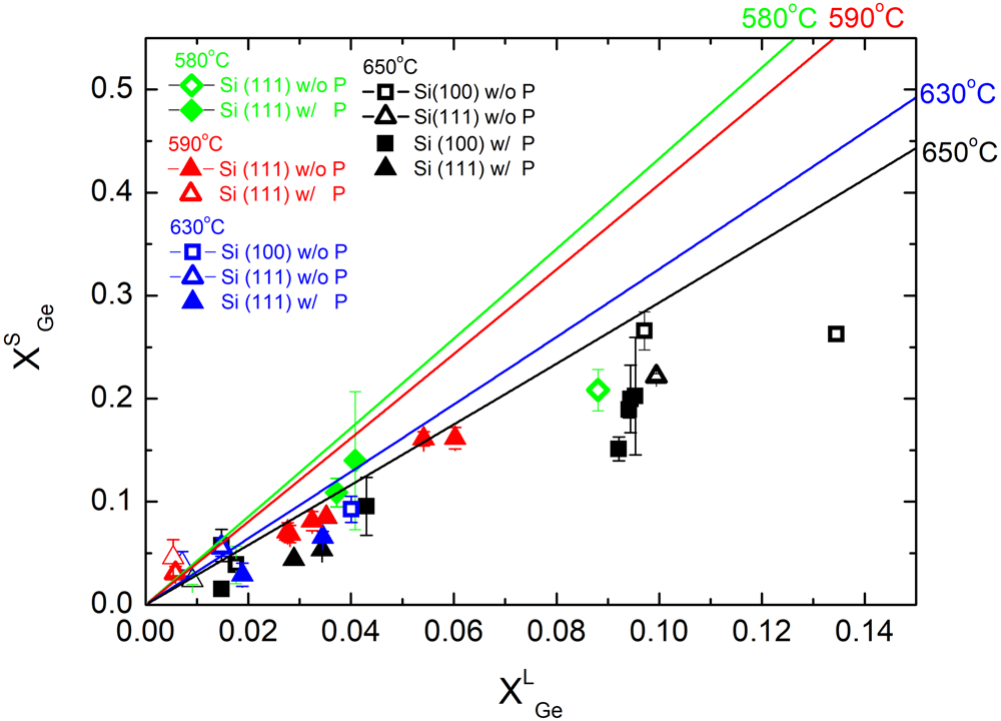
Phase diagram of the relation between Ge concentrations in solid (x) and in liquid (x_liquid_) for T_sat_ = 580 °C (green symbols), 590 °C (red symbols), 630 °C (blue symbols), and 650 °C (black symbols) based on our results. The open and solid symbols denote undoped and GaP-doped samples, respectively. The error bars denote the x variations between different crystals. Calculated relationships for T_sat_ = 580 °C (green line), 590 °C (red line), 630 °C (blue line), and 650 °C (black line) based on Eq. (1) are shown for comparison. It can be seen that the Ge concentrations of our SiGe crystals are always less than the calculated results and deviate from the model by more than 30%.

So far we have investigated the relations between Ge concentrations in solid (x), in liquid (x_liquid_), and T_sat_ for crystals grown at vacuum pressure 0.1 torr. The resulting experimental phase diagram is shown in Fig. 5. First, Fig. 5 shows a pronounce effect that higher x_liquid_ would lead to higher x. Second, lowering T_sat_ tends to yield higher Ge concentration in a crystal. Third, because no apparent deviations are found between Si (100) and (111) substrates, our results demonstrate that the LPE conditions are independent of Si substrates used. Finally, our data display large deviations from the calculated phase diagram. That is, the x measured is usually ~30% lower than the calculated values. In previous works, although agreements between experimental and calculated results were reported when using Bi as a solvent^20^, ~20% deviation was found when In was used as a solvent^18^. Because the calculated curves are obtained from parameters of Si-Ga or Ge-Ga binary systems with an assumption of negligible free energy of mixing, Borshchevsky and Fleurial have proposed that multicomponent interactions when introducing GaP doping would give substantial deviations from the ternary model^24^. Interestingly, although our result confirms the large effect of GaP on the solubility of Si and Ge in Ga solvent, Fig. 5 does not display pronounced differences between the undoped and highly-doped SiGe crystals, suggesting that the GaP plays a minor role in the phase diagram.

**Figure 6:**
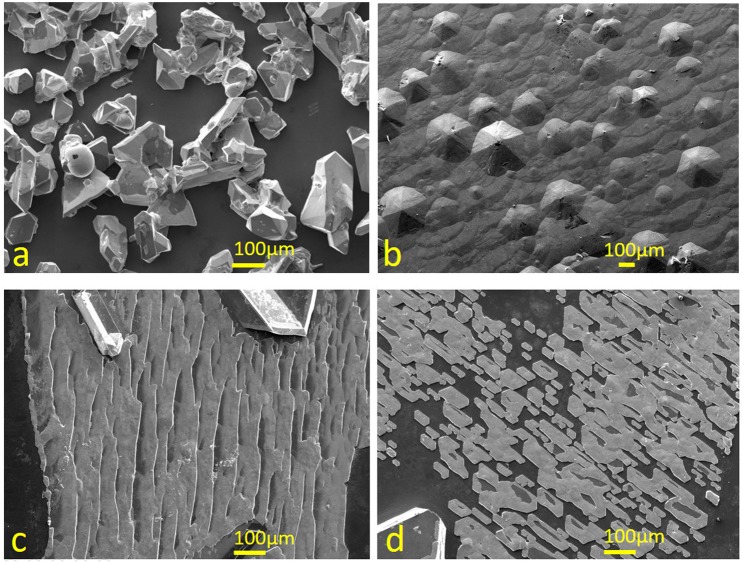
SEM images of different morphologies of high Ge concentration (**a**) crystals, (**b**) islands, (**c**) films, and (**d**) flakes commonly coexist with other SiGe crystals when LPE is conducted at pressure less than 10^−2^ torr.

When varying the H_2_ pressure during the growth, we have sometimes found various types of Ge precipitates coexist with SiGe crystals even though they are not anticipated from the phase diagram. As shown in Fig. 6(a–d), diverse morphologies varying from crystals, islands, films, to flakes are found at the surfaces when the pressure is less than 10^−2^ torr. Many of them are found to be Ge precipitation from EDS analyses, but they could be misidentified as the SiGe crystals in previous works. However, employing HF:H_2_O_2_:CH_3_COOH = 1:2:3 solutions to selectively etch away the unwanted Ge often yields porous materials^32^, implying poor selectivity. The pressure-dependent morphology changes have not been reported before, and their origin is likely due to some pressure-dependent thermodynamic quantities overlooked by earlier works.

**Figure 7:**
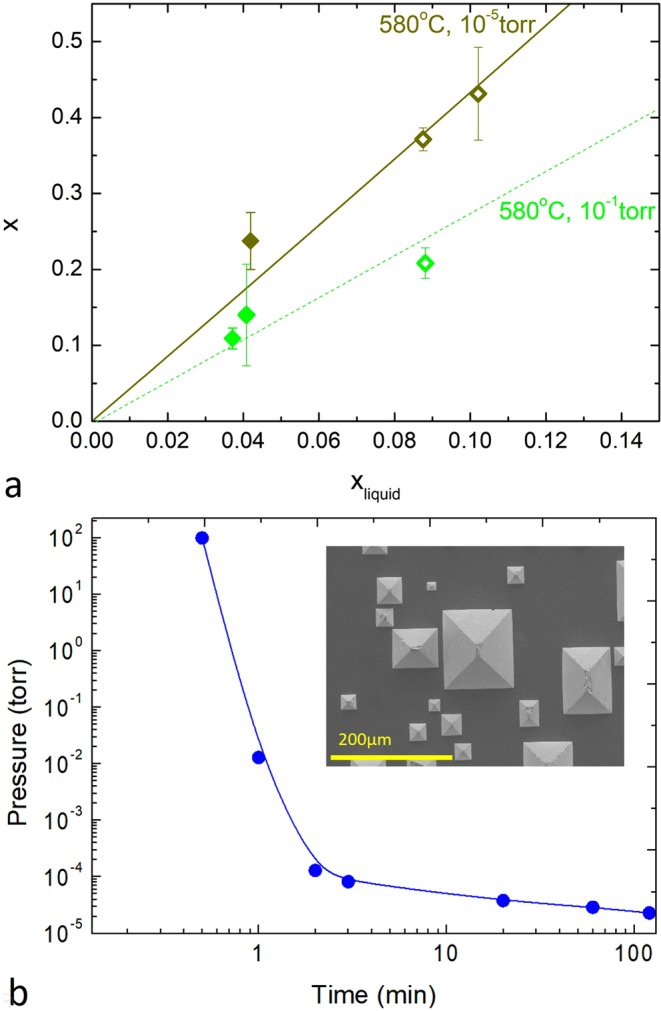
(**a**) Phase diagram for doped (solid symbols) and undoped (open symbols) SiGe crystals grown at T_sat_ = 580 °C under pressure 10^−5^ torr (dark yellow symbols) and 10^−1^ torr (green symbols), respectively. The dark yellow curve is the calculated result based on Eq. (1). The dotted green line is a fitted line for crystals grown at T_sat_ = 580 °C under pressure 10^−1^ torr. (**b**) Pressure vs. time when conducting pressure-induced LPE at T_sat_ = 580 °C. (Inset) A representative SEM image of the grown Si_0.2_Ge_0.8_ crystals.

To further elucidate the pressure dependent effects, we have conducted LPE at low vacuum pressures while keeping other conditions unchanged. Figure 7(a) shows the doped and undoped SiGe crystals grown at T_sat_ = 580 °C under pressure 10^−5^ torr and 10^−1^ torr, respectively. It can be seen that the crystals grown at 10^−5^ torr agree with the calculated curve, suggesting that the thermodynamic parameters used in previous works are applicable to high vacuum only. To directly demonstrate the pressure-induced LPE, Fig. 7(b) shows that we start by setting the initial pressure at 100 torr then quickly pump the pressure to 10^−5^ torr while keeping T_sat_ = 580 °C throughout the entire process. We have repeated the pressure-induced LPE with different initial Ge/Ga ratios. Interestingly, the resulting Si_1−x_Ge_s_ crystals always exhibit much higher Ge concentration than those crystals grown by conventional LPE mentioned above and x = 0.8 (shown in the inset of Fig. 7(b)) can be found. From the phase diagram shown in Fig. 1(b), x = 0.8 can be made only if T_sat_ > 775 °C and Bi is employed as the solvent. Thus, we demonstrate one key advantage of the novel pressure-induce LPE method. The pressure-induced LPE is a new concept rarely mentioned in previous works. Because pressure is much easier to control than temperature, it has been proposed to improve current LPE methods^33^. To our knowledge, our work is the first experimental demonstration of the method. It also points out the important role of regulating pressure during LPE processes.

In summary, we employ LPE to make GaP-doped Si_1−x_Ge_x_ (x = 0.05~0.25) crystals on Si (111) and (100) substrates. We successfully minimize unwanted concentration variations along the thickness of the grown SiGe crystals via keeping a constant cooling rate. Doping GaP into SiGe is found to affect the solubility of the system but it can be overcome by adding In into the Ga solvent and raising T_high_ of the furnace. The resulting highly-doped SiGe crystals display similar trends like those of undoped crystals in the phase diagram. Yet, we also find that the morphology and the Ge concentration of the grown crystals is sensitively dependent on the vacuum pressure during the growth. Significant deviations to the calculated phase diagram are found, which are attributed to an overlooked pressure-dependent effect. New pressure-dependent phase diagrams are plotted based on our results at 10^−1^ torr and 10^−5^ torr in Figs 5 and 7(a), respectively. Finally, we directly employ a pressure-induced LPE method to grow Si_1−x_Ge_x_ crystals and find the crystals exhibit much higher Ge concentrations than those grown by the conventional method.

## Methods

Experimentally, Si (111) and (100) substrates were cleaned using acetone, methanol and piranha solution (H_2_SO_4_:H_2_O_2_ = 3:1) followed by BOE etchant solution (HF/NH_4_F = 34.6% / 7.3%) etching to remove SiO_2_ and rinsing with deionized water. High purity Si (6N) and Ge (6N) powders were mixed with solvent Ga (6N) in a graphite crucible. A quartz tube was evacuated below 10^−5^ torr and then continuously purged with H_2_ at a set pressure using a precision leak valve during the entire process. After reaching the highest set temperature (T_high_ = T_sat_ + ΔT), the temperature was held for more than 2.5 h to homogenize the solution. We then carried out the LPE using either tipping boat or sliding boat methods. The growth process involved the dissolution of Si and Ge by the Ga melt, nucleation of the seeds at T_sat_ by rapidly cooling the substrate ΔT below T_high_, and separation of the growth layer from the melt. The growth time varied from 1.5 to 18 hours. Finally, the growth was terminated by removing the solution away from the substrate. After cooling the system to room temperature, excess Ga on the substrate was removed by adding Al foils in hot water. Similar procedures were used for GaP-doping as well, in which high purity P (5N) was mixed with the solvent at the beginning.

## Supplementary information

Supplementary information

## References

[1] Hsiao TK (2013). Observation of room temperature ballistic thermal conduction persisting over 8.3 micrometers in SiGe nanowires. Nature Nanotech..

[2] Hsiao TK (2015). Micron-scale ballistic thermal conduction and suppressed thermal conductivity in heterogeneously-interfaced nanowires. Phys. Rev. B.

[3] Chen J, Zhang G, Li BW (2009). Tunable thermal conductivity of Si_1−x_Ge_x_ nanowires. Appl. Phys. Lett..

[4] Shi LH, Yao DL, Zhang G, Li BW (2010). Large thermoelectric figure of merit in Si_1−x_Ge_x_ nanowires. Appl. Phys. Lett..

[5] Maire J (2017). Heat conduction tuning by wave nature of phonons. Sci Adv.

[6] Yazdani, S. & Pettes, M. T. Nanoscale self-assembly of thermoelectric materials: a review of chemistry-based approaches. *Nanotechnology* **29** (2018). 10.1088/1361-6528/aad673 30052199

[7] He J, Tritt TM (2017). Advances in thermoelectric materials research: Looking back and moving forward. Science.

[8] Huxtable ST (2002). Thermal conductivity of Si/SiGe and SiGe/SiGe superlattices. Appl. Phys. Lett..

[9] Cheaito R (2012). Experimental Investigation of Size Effects on the Thermal Conductivity of Silicon-Germanium Alloy Thin Films. Phys. Rev. Lett..

[10] Rowe DM, Shukla VS, Savvides N (1981). Phonon-Scattering at Grain-Boundaries in Heavily Doped Fine-Grained Silicon-Germanium Alloys. Nature.

[11] Liao BL (2015). Significant Reduction of Lattice Thermal Conductivity by the Electron-Phonon Interaction in Silicon with High Carrier Concentrations: A First-Principles Study. Phys. Rev. Lett..

[12] Garg J, Bonini N, Kozinsky B, Marzari N (2011). Role of Disorder and Anharmonicity in the Thermal Conductivity of Silicon-Germanium Alloys: A First-Principles Study. Phys. Rev. Lett..

[13] Koh YK, Cahill DG (2007). Frequency dependence of the thermal conductivity of semiconductor alloys. Phys. Rev. B.

[14] Vining, C. & Fleurial, J. F. In *the Tenth Int. Conf. on* Thermoelectrics. (ed. Rowe, D. M.) 1 (Barbrow Press, 1991).

[15] Zhu GH (2009). Increased Phonon Scattering by Nanograins and Point Defects in Nanostructured Silicon with a Low Concentration of Germanium. Phys. Rev. Lett..

[16] Shimura T (2015). Enhancement of photoluminescence from n-type tensile-strained GeSn wires on an insulator fabricated by lateral liquid-phase epitaxy. Appl. Phys. Lett..

[17] O’Reilly AJ, Quitoriano N (2018). Asymmetric, compressive, SiGe epilayers on Si grown by lateral liquid-phase epitaxy utilizing a distinction between dislocation nucleation and glide critical thicknesses. J. Cryst. Growth.

[18] Alonso MI, Bauser E (1987). Growth of Si_1−x_Ge_x_ on Silicon by Liquid-Phase Epitaxy. J. Appl. Phys..

[19] Hansson PO, Werner JH, Tapfer L, Tilly LP, Bauser E (1990). Liquid-Phase Epitaxy and Characterization of Si_1−x_Ge_x_ Layers on Si Substrates. J. Appl. Phys..

[20] Trah HP (1990). Liquid-Phase Epitaxy in the Ternary-System Si-Ge-Bi. J. Cryst. Growth.

[21] Healy SA, Young TL, Green MA (1991). Low-Temperature Growth of Silicon on Si1−xGax by Liquid-Phase Epitaxy. J. Cryst. Growth.

[22] Chan BO, Healy SA, Green MA (1992). Strained Si_1−x_Ge_x_ Layers Grown by Low-Temperature Liquid-Phase Epitaxy. Mater. Lett..

[23] Chen JX, Ernst F, Hansson PO, Bauser E (1992). Liquid-Phase Epitaxy of GeSi on (111) Si Substrates - Lattice Defect Structure and Electronic-Properties. J. Cryst. Growth.

[24] Borshchevsky A, Fleurial JP (1993). Growth of Heavily-Doped SiGe from Metallic Solutions. J. Cryst. Growth.

[25] Sembian AM (1998). Defect distribution and morphology development of SiGe layers grown on Si(100) substrates by LPE. Thin Solid Films.

[26] Fuller T, Konuma M, Zipprich J, Banhart F (1999). The critical thickness of silicon-germanium layers grown by liquid phase epitaxy. Appl. Phys. A.

[27] Sazaki G (2002). *In-situ* monitoring system of the position and temperature at the crystal-solution interface. J. Cryst. Growth.

[28] Schade, M. *et al*. Investigation of the chemical composition profile of SiGe/Si(001) islands by analytical transmission electron microscopy. *Appl. Phys. Lett*. **90** (2007).

[29] Lee SH, Healy SA, Young TL, Green MA (1990). Very-Low-Temperature Liquid-Phase Epitaxial-Growth of Silicon. Mater. Lett..

[30] Malmejac Y, Bonnier E, Desre P (1972). Contribution to Study of Ternary Ge-Si-Sb Phase-Diagram. Mem Etud Sci Rev Met.

[31] Thurmond CD, Kowalchik M (1960). Germanium and Silicon Liquidus Curves. Bcl] Syst. Tech. J..

[32] Hollander B, Buca D, Mantl S, Hartmann JM (2010). Wet Chemical Etching of Si, Si_1−x_Ge_x_, and Ge in HF:H_2_O_2_:CH_3_COOH. J. Electrochem. Soc..

[33] Mao X-J, Chan Y-C, Lam Y-L, Zhu J-Y, Shi JX (2000). New concept technology: pressure variation liquid phase epitaxy. Proc. SPIE.

