# Supplementary material for: Pressure-sensitive liquid phase epitaxy of highly-doped n-type SiGe crystals for thermoelectric applications
Source: Sci Rep. 2019 Mar 13;9:4362. doi: 10.1038/s41598-019-39786-y (PMC6416246; doi:10.1038/s41598-019-39786-y)
Supplement: Supplementary file 1 — Supplementary information [file 41598_2019_39786_MOESM1_ESM.docx]

Supplementary information for

**Pressure-sensitive liquid phase epitaxy of highly-doped n-type SiGe crystals for thermoelectric applications**

Hung-Wei Li and Chih-Wei Chang

**Cross sectional EPMA-WDS mapping of a grown crystal.** In addition to Fig. 3 of the main text, we have also performed EPMA-WDS measurements on the grown SiGe crystals, as shown in Fig. S1.


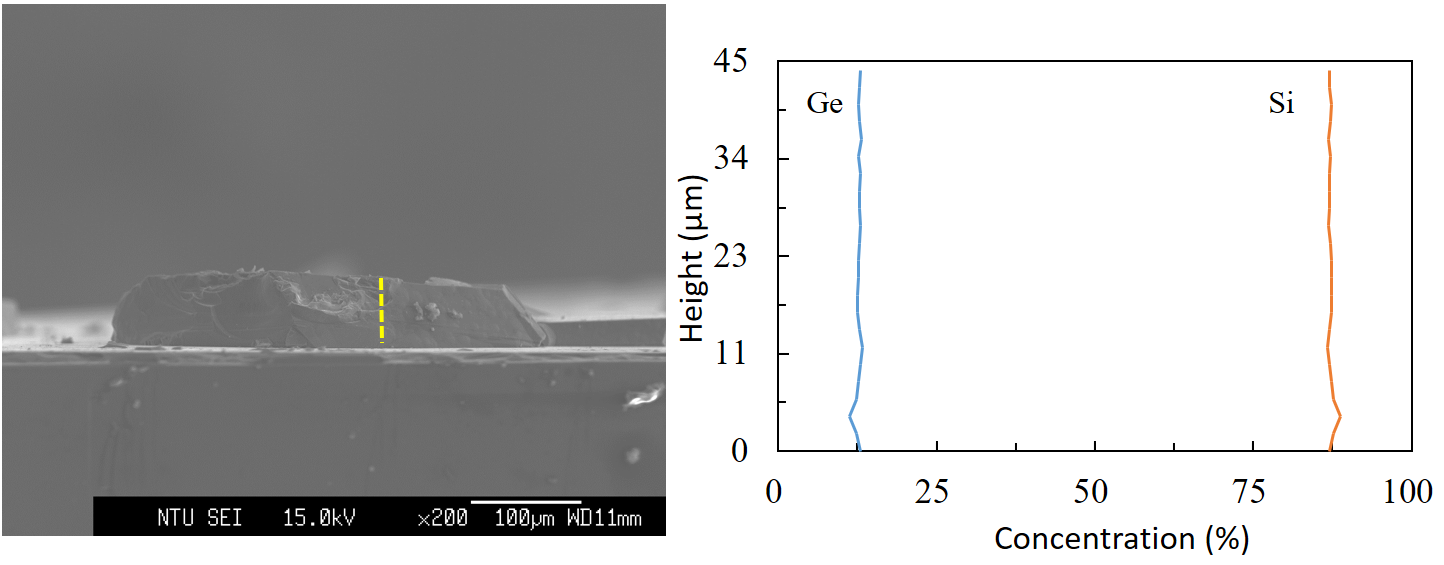


Fig. S1 (Left) Cross sectional SEM images of a SiGe crystal grown on Si (111) surface. (Right) The corresponding concentration profiles of Si (orange line) and Ge (cyan line) measured by EPMA-WDS, confirming that a cooling rate = 0^o^C/min can minimize concentration variations.
